# Acquisition of novel muscles enabled protruding and retracting mechanisms of female penis in sex-role reversed cave insects

**DOI:** 10.1098/rsos.220471

**Published:** 2023-01-11

**Authors:** Zixin Cheng, Yoshitaka Kamimura, Rodrigo L. Ferreira, Charles Lienhard, Kazunori Yoshizawa

**Affiliations:** ^1^ Systematic Entomology, School of Agriculture, Hokkaido University, Sapporo 060-8589, Japan; ^2^ Department of Biology, Keio University, Yokohama 223-8521, Japan; ^3^ Ecology and Conservation, Federal University of Lavras, CEP 37200-900 Lavras (MG), Brazil; ^4^ Geneva Natural History Museum, 1211 Geneva 6, CP 6434 Switzerland

**Keywords:** female genitalia, gynosome, homology, *Neotrogla*, muscle

## Abstract

Brazilian sex-role reversed cave insects (genus *Neotrogla*) have a striking structure called the gynosome (or female penis), which deeply penetrates male vagina-like genitalia during copulation to receive nutritious semen. However, the protruding and retracting mechanisms of the female penis, including their evolutionary origin, are poorly understood. By using micro-computed tomography (µCT), we compared the genital morphology and musculature between species with a gynosome and others lacking this structure. As a result, we discovered two groups of muscles related to the protrusion and retraction of gynosomes. These muscles were also observed in species with non-protrusible prepenis. This suggests that evolution of these muscles preceded the acquisition of the protruding function of the gynosome, originally having a putative stimulatory function to receive nutritious semen. This intermediate stage probably allowed for the reversal of genital functions.

## 1. Introduction

To date, the occurrence of a female penis has only been found in a small tribe of cave insects, Sensitibillini (Psocodea: Trogiomorpha: Prionoglarididae) [1,2]. In the genus *Neotrogla*, females have a penis-like intromittent organ called the gynosome, which penetrates deeply into the male vagina-like cavity during copulation to receive voluminous and nutritious semen [1]. The origin of the female penis is still an insufficiently understood phenomenon. Frequently, there is a significant gap between ancestral and derived morphology, and how the intermediate condition functioned is poorly understood [3]. The reversals of male and female genital function require complete correlation between the sexes, and the evolution of such anomalies must be strongly constrained [2]. The oligotrophic cave environment [4] and seminal nuptial gift [1] are thought to be the key factors facilitating female competition and, as a consequence, the origin of the female penis [5,6]. In addition, females of *Neotrogla* have multiple sperm storages (twin insemination slots with switching valve to receive twice the amount of nutritious seminal gift at the same time) [7]. This unique feature renders female–female competition more intense, favouring the evolution of a female penis [2,6,8].

There are also factors relaxing the constraints against genital reversal [2,9–11]. Males of Psocodea usually do not have a penetrative penis, and seminal transfer is achieved by tight contact between the flat female spermapore plate (a plate surrounding the opening of the spermathecal duct, which leads to the sperm storage organ) and the non-bulging opening of the male seminal duct. The spermapore plate is hooked and pulled out from the female genital chamber toward the male seminal duct by using penile or non-penile structures of the male terminalia [2,10,12]. A genus in the same suborder as Sensitibillini (Trogiidae: *Trogium*), the female's spermapore plate is modified into a small tubercle. During copulation, the male grasps the tubercle by using the parameres so that it shallowly penetrates the male genitalia [2,10]. The female tubercle is widely observed in Trogiomorpha, including in a close relative of Sensitibillini (the genus *Speleketor*, belonging to Speleketorinae together with Sensitibillini), which is thought to exhibit a preadaptive condition, relaxing the constraint against the evolution of a deeply penetrating female penis [11,13,14]. In this sense, the female penis may also be regarded as an exaggeration of the tubercle already derived in Trogiomorpha.

However, the origins of the protrusion and retraction mechanisms of the gynosome, including its function in transitional evolutionary stages, are completely unknown to date. In this study, we reconstructed three-dimensional models of female genital structures of *Neotrogla* using the micro-computed tomography (µCT) technique and compared musculature related to the gynosome with the muscles of female genitalia in other psocodeans to detect their homology and to identify novelties related to the mechanical function of the female penis.

## 2. Material and methods

A female not in copula (Voucher ID, Hokkaido University: S8KY01) and a copulating pair (S8KY05) of *Neotrogla curvata* Lienhard & Ferreira, 2013 [4] and a female not in copula of *N. brasiliensis* Lienhard, 2010 (S8KY03) [15] were used for µCT examination. Samples were fixed with hot water (approx. 80°C). Dehydration was conducted in ascending order with 80–100% ethanol before drying the specimens at the critical point (EM CPD300, Leica, Wetzlar, Germany) avoiding serious organ shrinkage. Samples were scanned using the synchrotron µCT at the BL47XU [16] beamline of the SPring-8 (Hyogo, Japan) using a stable beam energy of 8 keV in absorption-contrast mode. The tomography system consists of a full-field X-ray microscope with Fresnel zone plate optics [17]. We used semi-automatic segmentation algorithms based on grey-value differences in ITK-SNAP software [18] to obtain three-dimensional representations. µCT-based morphological data of three species of the suborder Trogiomorpha, *Prionoglaris stygia* Enderlein, 1909 (S8KY08) [19], *Psyllipsocus clunjunctus* Lienhard, 2013 (S8KY10) [13], *Lepinotus reticulatus* Enderlein, 1904 (S8KY12), of which *P. stygia* is a member of Prionoglarididae together with Sensitibillini, were from the previous study [14].

Morphological information of *Afrotrogla oryx* Lienhard, 2007 (having female penis) [20], *Sensitibilla etosha* Lienhard & Holusa, 2010 (having prepenis) [21] and *Speleketor irwini* Mockford, 1984 (lacking female penis but with tubercule) [22] were obtained by using a compound light microscopy observation (Zeiss Axiophot: Oberkochen, Germany). A photograph of *S. etosha* was taken with an Olympus E-M5 (Tokyo, Japan) attached to a Zeiss Axiophot.

**Figure 1.**
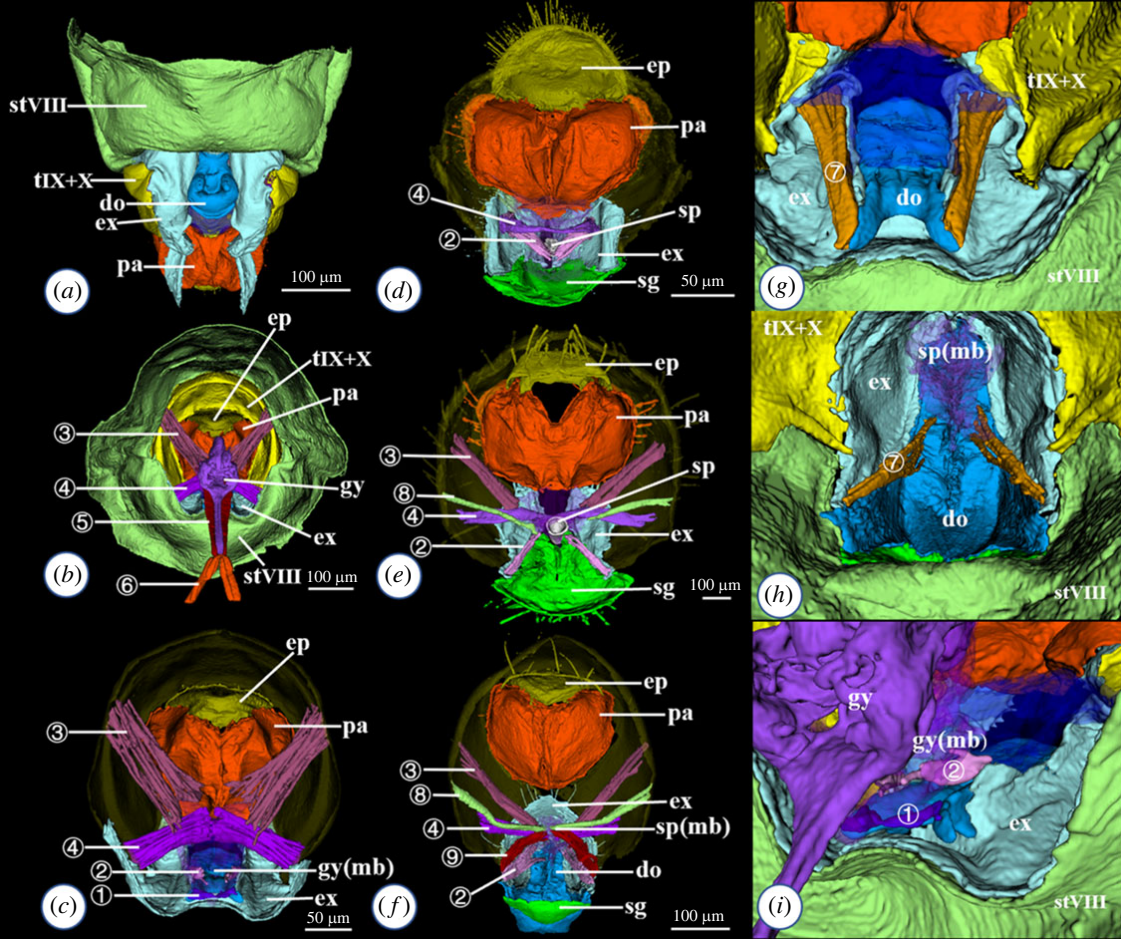
Female terminalia of the trogiomorphan species: *Neotrogla curvata* (*a–c*), *Prionoglaris stygia* (*d*), *Psyllipsocus clunjunctus* (*e*), *Lepinotus reticulatus* (*f*): (*a*) ventral external view; (*b–f*) internal view ((*c*): gynosome omitted); (*g–i*) close-up of the gonapophyses and related structures, internal view: *Neotrogla curvata* (*g,i*); *Lepinotus reticulatus* (*h*). Muscle numbers (for terminology see [13]): (1) dosp01; (2) exsp01; (3) spIX01; (4) spIX02; (5) muscle 5; (6) muscle 6; (7) doex01; (8) spIX03; (9) spVIII01. do = dorsal valve; ep = epiproct; ex = external valve; gy = gynosome; mb = membrane; pa = paraproct; sg = subgenital plate; sp = spermapore plate; st = sternum; t = tergum.

**Figure 2.**
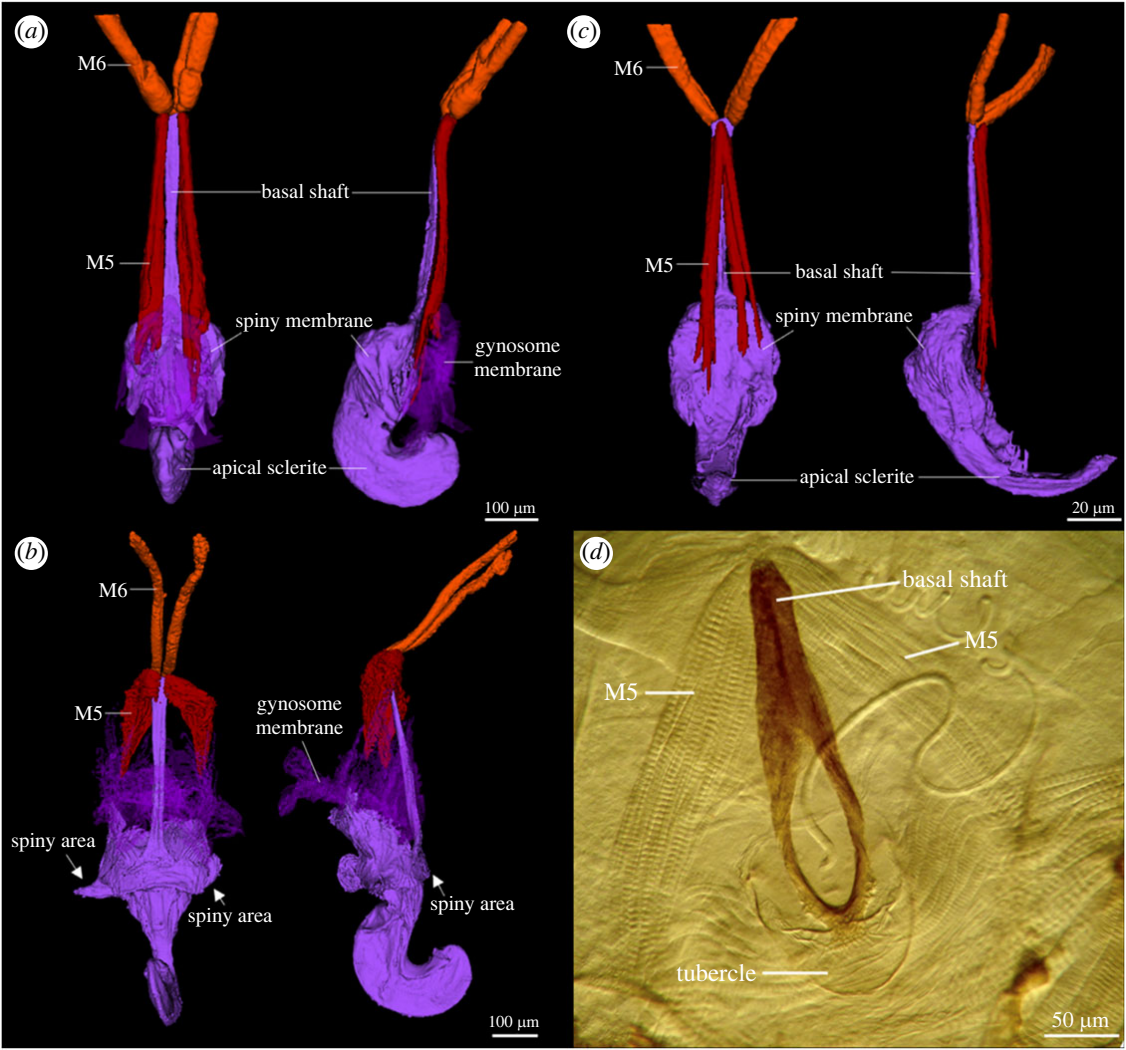
(*a*, *b*) Gynosome of *Neotrogla curvata* (left: ventral view; right: lateral view): (*a*) uncopulated state; (*b*) copulated state. (*c*) Gynosome of *Neotrogla brasiliensis* in uncopulated state (left: ventral view; right: lateral view). (*d*) Prepenis and its associated muscles of *Sensitibilla etosha* (ventral view). M5 = muscle 5; M6 = muscle 6.

The following terminology was adopted for the gynosomal structures [1]: apical sclerite—well-sclerotized structure bearing the opening of the spermathecal duct [1] (figures 1*b* and 2*a*); spiny membrane—the membrane surrounding the apical sclerite and bearing many spines anchoring males during copulation; gynosome membrane—the additional membrane extending from the ventral region of the spiny membrane and bearing the muscle attachments (figure 2*a*); basal shaft—the internal apodeme extending from the anteroventral margin of the apical sclerite (figure 2*a*).

The ancestral state of the genital characters was estimated based on a molecular phylogenetic tree (including the branch length) of Prionoglarididae as estimated previously ([8], topology including branch length available as electronic supplementary material, Data at Dryad) using the likelihood method as implemented in Mesquite v. 3.5 [23]. Two evolutionary models, Mk1 (one-parameter model) and AsymmMK (two-parameter model, giving different rates for gain and loss of a character), were applied, and better fitting model was selected by Akaike information criteria (AIC) [24]. Taxa lacking morphological information were trimmed from the tree. The morphological information of the outgroups were from the µCT-based character analyses conducted by Cheng & Yoshizawa [14]. However, for most outgroup species, µCT data were not available. Therefore, morphological information obtained from the same family member were adopted for the terminal taxa.

## 3. Results and discussion

Among genital structures observed in females, the epiproct and paraproct (structures surrounding the anus) of *Neotrogla* showed little morphological differences from other psocodeans. By contrast, there were unique modifications in gonapophyses (which comprise the ventral, dorsal and external valves homologous to the appendages of the eighth and ninth abdominal segments: also termed ovipositor valves), the spermapore plate (sclerite surrounding the opening of the sperm storage organ), and the subgenital plate (posterior extension of the eighth abdominal sternite, covering the base of the gonapophyses and entire spermapore plate) [14,25]. The gynosome may have been homologous to one of these structures.

In *Neotrogla*, we found two structures that may be homologous to gonapophyses. One was a pair of well-developed valves forming the ovipositor (figure 1*a*: light blue). Comparisons of the external morphology (positional congruence) and musculature (see below for the additional muscles supporting the interpretation) strongly suggest that the ovipositor valves of *Neotrogla* are homologous to the external valves of the gonapophyses, as in other trogiomorphan species [14,15]. The other structure is an undivided lobe situated between the bases of the external valve (figure 1*a*: deep blue). In previous studies, this structure was interpreted as a part of the subgenital plate [4,15]. However, a muscle originating from the base of the external valve is inserted into the anteroventral margin of this structure (figure 1*g*: muscle 7). No muscles connecting the gonapophyses and the subgenital plate have been found in other psocodeans [14]. In addition, this lobe-like structure is located posterior to the external valve (ninth segment) so that this structure is not homologous to the subgenital plate (eighth sternum). The only muscle that connects two structures in this way is doex01 (connecting dorsal and external valves) (figure 1*h*) [14]. Therefore, it is most likely that this lobe-like structure corresponds to the medially fused pair of dorsal valves of the gonapophyses [14]. The distribution of the doex01 muscle within Trogiomorpha is highly heterogeneous [14] but, because the dorsal valve is greatly reduced in the most species of this suborder, multiple reductions of this muscle due to the functional reduction of the dorsal valve within Trogiomorpha are probably likely. Thus, homology between the gynosome and the gonapophyses can be ruled out, and the subgenital plate of *Neotrogla* is considered to be reduced.

Instead, most of the muscles associated with the gynosome can be homologous with those associated with the spermapore plate of other psocodeans [14]: muscle 1—dosp01 (connecting the dorsal valve and spermapore plate: figure 1*c*, *i*), muscle 2—exsp01 (external valve and spermapore plate: figure 1*c–f*), muscle 3—spIX01 (spermapore plate and 9th tergum: figure 1*c*, *e*, *f*), and muscle 4—spIX02 (spermapore plate and 9th tergum: figure 1*b–f*) (table 2). Although no muscle corresponding to dosp01 (muscle 1) was found in other species of the suborder Trogiomorpha (probably related to the reduction of the dorsal valves in this suborder), most species in the other psocodean suborders have this muscle [14]. Sharing of these muscles strongly suggests homology between the spermapore plate and the gynosome. Furthermore, presence of the opening of the spermathecal duct strongly supports homology between the spermapore plate and the apical sclerite of the gynosome. The muscles associated with the spermapore plate function to restore the plate and the surrounding membrane after copulation in psocopterans without female penis [14]. The above-mentioned gynosome muscles are not directly associated with the sclerotized part. Therefore, they most likely act to restore the gynosomal spiny membrane at the right position after copulation (figure 1*b, c*). Of them, the muscles 3 and 4 of *Neotrogla* are especially thicker than those of the species lacking the gynosome (figure 1*b–f*), suggesting that it needs more muscle power to restore the enlarged and complicated gynosomal spiny membrane.

In addition to the muscles mentioned above, we found two pairs of muscles directly attached to the gynosomal basal shaft in both species of *Neotrogla*. Comparisons of the muscles of individuals in copulating and uncopulating states showed that these muscles function as protractors and retractors of the gynosome and are key factors for its function. The muscle 5 (figure 2*a–c*) originates on the gynosome membrane very close to dorsal valves and is inserted on the anterior end of the basal shaft. Based on the absence of the subgenital plate in *Neotrogla*, the possibility of the basal shaft of the gynosome, at least in part, involved the element of the subgenital plate need to be discussed. There are two groups of muscles (16 (sgVIII01) or 17 (sgVIII02) in Cheng & Yoshizawa [14]) connected to subgenital plate in Psocodea, both originated from the subgenital plate and inserted to the eighth abdominal segment. Most importantly, the gynosome including its basal shaft is located posterior to the external and dorsal valves (appendages of the ninth segment), although the subgenital plate represents the posterior extension of the eighth sternum. Therefore, the homology between the subgenital plate and the gynosomal basal shaft can be ruled out, and the basal shaft should be interpreted as the internal extension of the anterior margin of the spermapore plate. The muscle 5 is originated from the tip of the basal shaft and is inserted to the spermapore plate membrane, which is also located posterior to the external and dorsal valves (= ninth segment). Because both the origin and insertion sites of muscle 5 are on the ninth segment, the homology of this muscle with the other subgenital plate muscles (sgVIII01 or 02, both are on eighth segment) is also unlikely. No other muscle that can be homologous to the muscle 5 is attached to the spermapore plate of other psocodeans, and the muscle 5 is here interpreted as a muscle newly achieved in Sensitibillini. The muscle 6 may possibly corresponds to spIX03 (muscle 8 of figure 1*e,f*: originated from the spermapore plate and inserted to the ninth tergite) of other psocodeans, because it is absent in *Neotrogla*. The muscle 6 of *Neotrogla* is inserted to the internal organ, which is quite different from the condition of spIX03 of other psocodeans. Therefore, this homology interpretation is probably less likely, but the positional change and acquisition of a novel functional of the spIX03 muscle in *Neotrogla* cannot be ruled out.

The pair of the muscle 5 were contracted in the copulated state (i.e. gynosome protruded) and were relaxed in the uncopulated female. Therefore, the pair of the muscle 5 is interpreted as a protractor of the gynosome. The muscle 6 (figure 2*a–c*) originates on an internal organ (specific attachment site not detected) and is inserted into the anterior end of the basal shaft. These muscles were relaxed in the copulated state but were contracted in the uncopulated female (i.e. gynosome stored in the abdomen) (figure 2*a*,*b*). Therefore, the pair of muscle 6 is interpreted as a retractor of the gynosome. These observations clearly showed that the development of the basal shaft and two groups of muscles attached to it are important functional derivations to achieve the protruding and retracting movements of the gynosome.

**Figure 3.**
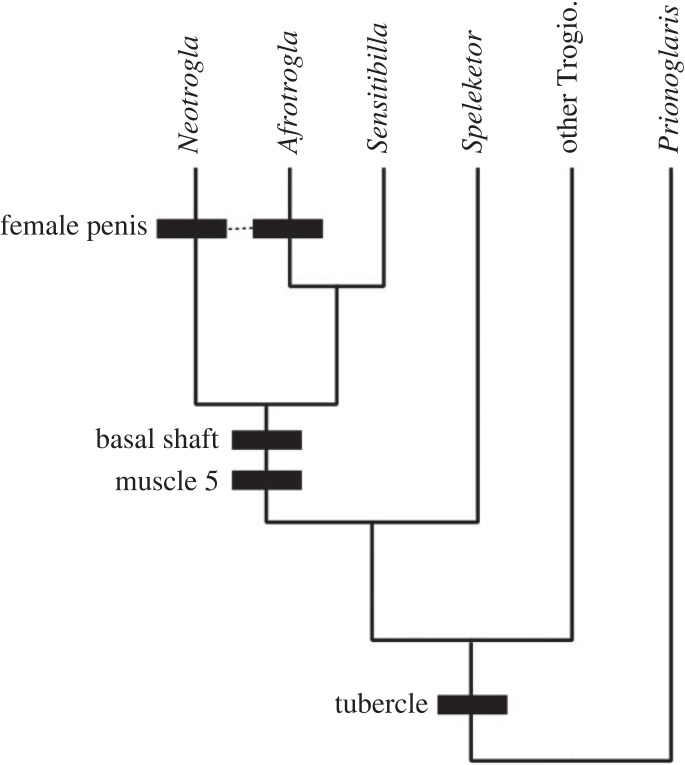
Phylogeny of Trogiomorpha, and the ancestral state reconstruction of characters related to the evolution of the gynosome. Black bar indicates the branch where the derived character evolved. The ‘other Trogio.’ includes genera *Dorypteryx* (Psyllipsocidae), *Rhyopsocus* (Psoquillidae), Cerobasis (Trogiidae), *Soa* and *Echmepteryx* (Lepidopsocidae).

Key questions remain regarding the origins of the shaft and these muscles and their function in a primitive stage. To elucidate these questions, we estimated the ancestral state of some characters related to the female penis (figure 3). For characters 1–3, the Mk1 model (same parameter for gain and loss of a character) was interpreted as a better fitting model. By contrast, AsymmMk model (different parameters for gain and loss of a character) was interpreted as a better fitting model for character 4 (electronic supplementary material, table). Under this model, a single origin of the female penis at the common ancestor of the Sensitibillini (59.1% presence versus 40.9% absence) and secondary loss of the female penis in *Sensitibilla* were regarded as slightly more likely. However, this interpretation obtained under the AsymmMK model is unlikely in some aspect. Most importantly, the gynosome of *Afrotrogla* and *Neotrogla* is significantly different morphologically, and they are considered to have been formed by enlargement of different parts of the prepenis [8]. In addition, under the AsymmMK model, evolutionary rate for the loss of the female penis (9.3) was estimated to be much higher than the gain (1.3) but, once the female penis evolved, its secondary loss (equivalent to the secondary loss of the male penetrative organ, which is extremely rare in animals with internal fertilization [26]) must be strongly constrained. Therefore, independent origins of the female penis between the genera *Afrotrogla* and *Neotrogla*, as obtained under the Mk1 model, is here accepted as a more likely assumption [8] (figure 3 and electronic supplementary material, figure), although uncertainty still remains regarding the single or multiple origins of the female penis. To clarify this question, discovery of new Sensitibillini taxa having or lacking the female penis is needed, because the present phylogenetic tree includes all the Sensitibillini genera described to date.

The basal shaft (character 2) has been observed in all genera of Sensitibillini (*Afrotrogla*, *Neotrogla* and *Sensitibilla*) [8], and muscles homologous to muscle 5 (character 3) have been observed in *Sensitibilla* (figure 2*d*) [2] (the presence/absence of this muscle is unknown from *Afrotrogla*) (table 1). The ancestral state reconstruction of these characters suggested that the origin of the shaft and muscle 5 can be traced back to the common ancestor of Sensitibillini [8], possibly preceding the origins of female penis (figure 3 and electronic supplementary material, figure). The previous morphological analyses strongly suggested that the spermapore plate of *Sensitibilla* (called prepenis) does not form a protruding structure, different from *Neotrogla* and *Afrotrogla* [8]. Therefore, the shaft and associated muscles in the non-protruding prepenis of *Sensitibilla* are expected to have different preceding functions.

**Table 1.** Distribution of selected characters related to the evolution of the gynosome among the subfamily Speleketorinae.

	tubercle	basal shaft	muscle 5	female penis
*Speleketor*	yes	no	no	no
*Sensitibilla*	yes	yes	yes	no
*Afrotrogla*	yes	yes	?	yes
*Neotrogla*	yes	yes	yes	yes

**Table 2.** Muscles of the female genitalia and their sites of origin and insertion.

	origin	insertion
dosp01	dorsal valve	spermapore plate
exsp01	external valve	spermapore plate
spIX01	tergum IX	spermapore plate
spIX02	sternum IX	spermapore plate
M5	gynosome membrane	basal shaft
M6	internal organ	basal shaft
doex01	dorsal valve	external valve
spIX03	segment IX	spermapore plate
spVIII01	sternum IX	spermapore plate

The spermapore plate is modified into a small tubercle in many trogiomorphan species (character 1), including *Sensitibilla* (figures 2*d* and 3)*,* and during copulation, this tubercule slightly penetrates the male genitalia [2,10]. In addition, active competition for seminal nutrition by females has also been observed in other trogiomorphans [2,27]. The most likely explanation is that the modification of the spermapore plate toward the female penis was promoted by sexual selection to receive more seminal nutrition [2,6]. The shaft and associated muscles in the non-protruding prepenis of *Sensitibilla* are also expected to have functions increasing the female's benefit, such as stimulation of the male mate in order to receive more nutritious semen.

Our present examinations showed that many modifications are involved in the evolution of the functional female penis, including the modification of the pre-existing structures (e.g. from the tubercle to the gynosomal apical sclerite) or development of completely novel ones (e.g. the basal shaft and muscles 5/6). Most of these structures are thought to have different functions at intermediate conditions. To identify a possible trigger of the unique evolution of the female penis, biomechanical analyses of the *Sensitibilla* preprenis will be a key for understanding the evolutionary history.

## Data Availability

Raw scanned images and NEXUS formatted data used for the ancestral state estimations are available from the Dryad Digital Repository: https://doi.org/10.5061/dryad.rn8pk0pcv [28]. Data are available from Figshare [29] and are provided in the electronic supplementary material [30].
